# Development of non‐contact foreign body imaging base on photoacoustic signal intensity measurement

**DOI:** 10.1002/acm2.14230

**Published:** 2023-11-28

**Authors:** Andreas Setiawan, Chia‐Yi Huang, Mitrayana Mitrayana

**Affiliations:** ^1^ Department of Physics Universitas Kristen Satya Wacana Salatiga Indonesia; ^2^ Department of Applied Physics Tunghai University Taichung Taiwan R.O.C; ^3^ Department of Physics Faculty of Mathematics and Natural Sciences Universitas Gadjah MadaSekip Utara Bulaksumur Yogyakarta Indonesia

**Keywords:** foreign body, photoacoustic imaging, Rosencwaig‐Gersho theory

## Abstract

**Background:**

It is challenging to visually identify tiny and concealed foreign objects within the body due to their small size and subcutaneous location while they can cause infections.

**Methods:**

A non‐contact photoacoustic system based on Rosencwaig‐Gersho photoacoustic theory and dual modulator method is developed for detecting foreign objects in meat.

**Result:**

The experiments conducted validate the successful development of this measurement technique with 10 μm spatial resolution and its corresponding mathematical model, demonstrating an 11% Mean Absolute Percentage Error (MAPE) in comparison to the experimental results. Dual modulator successfully regulates laser energy at MPE limit.

**Conclusion:**

The utilization of non‐contact photoacoustic signal intensity measurements enables the identification of foreign objects within the body. Further, the application of mathematical modelling can validate the measurement outcomes. These findings serve as a foundation for creating an affordable and straightforward foreign body detector.

## 1 INTRODUCTION

A foreign body refers to an object lodged within the human body unintentionally. Such incidents can transpire in different organs throughout the body.^1^ In the case of subcutaneous foreign bodies, they typically arise when an object penetrates beneath the skin.^2^, ^3^ Objects composed of metal, such as shrapnel, syringes, fish hooks, and similar items, heighten the risk of infection since they serve as potential entry points for bacteria and other pathogens.

The current imaging techniques employed for foreign body detection encompass x‐ray computed tomography (CT), magnetic resonance imaging (MRI), and ultrasound (US).^4^, ^5^ X‐ray CT is commonly utilized for mass screenings to identify radiopaque foreign objects. Nonetheless, it is unsuitable for detecting radiolucent materials such as wood, fabric, and plastic.^6^, ^7^, ^8^ MRI is increasingly being used as an alternative; however, it poses risks when detecting metal fragments, which can endanger the patient's safety.^9^ Ultrasound, on the other hand, is widely adopted in clinical settings due to its real‐time imaging capability, absence of ionizing radiation, and affordability. Nevertheless, it exhibits low sensitivity and specificity in detecting small foreign bodies, and distinguishing between vital and non‐vital tissues in wounds proves challenging.^10^

Photoacoustic (PA) imaging can map blood vessels and organ function without external contrast material with high resolution and can map much deeper than other optical technologies.^11^, ^12^, ^13^ Imaging of the wound area can be performed in real time. The ability to adjust the wavelength helps identify the type of debris.^14^ In general, photoacoustic imaging detects objects by measuring the time of flight (TOF). Numerous photoacoustic systems use the principle of measuring in the time domain, which measures the magnitude of the peak.^15^ In PA imaging, short pulses of laser light are transmitted to irradiate the tissue, and are absorbed in the tissue, generating acoustic signals due to the thermo‐elastic expansion. If working above the audio frequency, then these ultrasound signals can be received by a conventional US transducer to reconstruct PA images.^16^ Basically, PA images can be thought of as ultrasound images whose contrast does not depend on the mechanical properties and elasticity of the tissue, but on its optical properties, specifically optical absorption.^17^ Instrumentationally, TOF measurement requires an acoustic sensor that works in the ultrasonic region (due to the high speed of sound in the material and the short distance travelled). This is why a lock‐in amplifier is always used in every TOF‐based measurement. In some measurements resonance tubes are used to overcome this, a resonator cell is used to improve SNR up to 100 points.^18^ This closed cell will effectively suppress noise outside the cell resonance frequency. The method used is to adjust the photoacoustic modulation frequency to work at the cell resonance frequency. With this method, the photoacoustic signal generated will work at the resonant frequency. As a result, the cell will resonate and function as a band‐pass filter, amplifying the photoacoustic signal while suppressing other noise. The challenge of this method is that the object must be located within a closed cell. This places dimensional constraints on the objects to be measured.

On the other hand, the use of ultrasonic frequencies in PA measurement presents new challenges. Ultrasonic sensors generally require contact with the object surface through a coupling medium to improve the impedance between the object and the sensor.^19^, ^20^ Consequently, this measurement faces some challenges on certain objects such as small object areas,^21^ uneven surfaces^22^ and infections.^23^ Some experiments have reported the use of audio frequencies to overcome this.^24^ In the report, a modulation frequency of 19 kHz is used so that audio microphones can be used. The experiment demonstrates the imaging of micro objects (fibre and hair) with a lateral resolution of 135 μm. However, the method developed is intended for surface objects, and has not been tested for sub‐surface conditions. The use of audio frequencies to detect subsurface objects has also been reported.^25^ In the report, a photoacoustic amplitude measurement method is used on a metal object coated with paint. Because the object is made of metal material, the range of laser power used is wider without damaging its physical condition. A different effect will occur when this method is applied to sensitive biological objects, where excessive laser power will damage the physical condition of the object. Since the laser power will be directly proportional to the photoacoustic amplitude produced, a strategy is needed to obtain optimal results. The strategy used was to adjust the laser wavelength and design a dual modulator to optimise the absorption on the object without exceeding the allowable laser power limit. This is done by selecting the laser working area according to the chromophore absorption of the biological object to be used, namely, chicken meat. In this research, a 980 nm laser was chosen, which is quite close to the chicken meat chromophore derived from O‐H bonds at 970 nm.^26^ The laser energy control method developed must be compatible with the photoacoustic method to be used. For this reason, the pulse width modulation (PWM) method was chosen to reduce the laser exposure time on the object. However, because the thermal diffusion length is also related to the laser modulation frequency, dual PWM modulations are carried out.

Related to the resolution of PA microscopic imaging, in general the methods often used in photoacoustic imaging are optical‐resolution photoacoustic microscopy (ORPAM) and acoustic‐resolution photoacoustic microscopy (ARPAM). ORPAM uses a tight optical focus to obtain the smallest possible exposure point. It can have a high resolution, as it is affected by the wavelength of the laser used. However, this advantage leads to shallow penetration due to light diffusion interference in the sub‐surface area. Meanwhile, ARPAM uses tight‐focus acoustic transducers to obtain the smallest possible measurement points. ARPAM provides better penetration capability because acoustic propagation is better than the optical one in sub‐surface areas due to less scattering of ultrasound. However, the minimum focus size limitation of acoustic transducers results in lower resolution than ORPAM. Both ORPAM and ARPAM methods perform TOF measurements. Therefore, achieving high resolution requires precision timing instruments and a wide sensor bandwidth (to detect narrow photoacoustic pulses). Conventional photoacoustic techniques have a significantly reduced signal‐to‐noise ratio (SNR) and PA signal amplitudes in the order of nV, making measurement challenging. Lock‐in amplifiers are used to overcome this. However, these devices are still expensive and bulky, making them impractical to use in the field.^27^, ^28^

Based on this background, this research uses a different measurement method. In this research, a photoacoustic signal amplitude intensity measurement is used. This idea is based on a number of successful test reports of measuring multilayer thin films with nm accuracy using modulated lasers and photothermal methods.^29^ However, since the TOF measurement method is no longer used, another breakthrough is needed for imaging subsurface objects. In this research, a photoacoustic imaging technique based on Rosencwaig‐Gersho theory (RG Theory) is developed.^30^, ^31^, ^32^ Using this theory, the photoacoustic modulation frequency can be used as a parameter to detect the presence of subsurface objects through the measurement of the resulting photoacoustic signal intensity. One of the techniques used is to reduce the attenuation of acoustic wave propagation by using audiosonic working frequency (below 20 kHz). The use of audiosonic frequencies will improve the acoustic impedance ratio between meat and air thus increasing the transmission coefficient of acoustic waves. In addition, audiosonic measurements can use audio devices that are easier to find. This research showcases the outcomes of developing foreign body imaging utilizing the proposed theory, along with the creation of its corresponding mathematical model.

## 2 MATERIAL AND METHODS

### 2.1 Photoacoustic signal intensity and subsurface imaging

**FIGURE 1:**
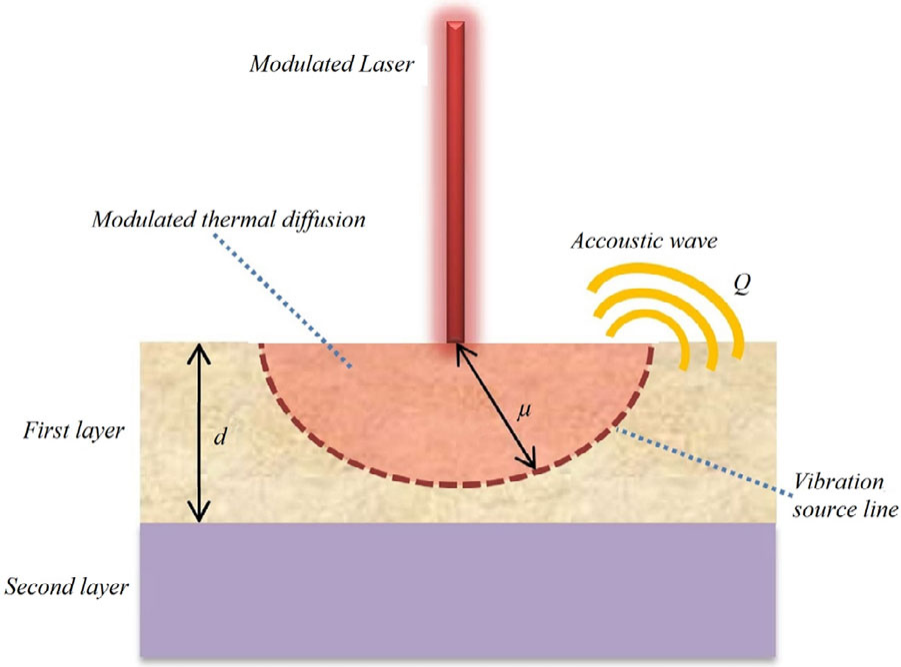
Illustration of acoustic wave production using the Rosencwaigh‐Gersho piston theory. A laser modulated with frequency *f_mod_* will cause the appearance of a thermal diffusion area with radius *μ*.

In this experiment, the Rosencwaig‐Gersho piston theory is used as the basis for imaging subsurface foreign bodies. Two different materials are used (meat as the first layer and foreign body as the second layer) as shown in Figure 1.

Based on this theory, the condition required for photoacoustics to penetrate opaque objects is

(1) μ>d

where *μ* and *d* are the thermal diffusion length and layer thickness, respectively. In this case the thermal diffusion length for modulated lasers can be calculated using the relation in the equation

(2) $$\mu ={\left(\frac{\alpha_{1}}{\pi \;f_{\mathrm{mod}}}\right)}^{1/2},$$

where *α_1_* and *f_mod_* are the thermal diffusivity of the first layer and the laser modulation frequency, respectively.

Using the illustration in Figure 1, a laser that interacts with the surface will cause the appearance of a thermal diffusion region below it. However, because the laser beam is modulated, the spread of this area will stop in the area with a radius of *μ*. As a result of this heating, the thermal diffusion area will experience expansion more dominantly than the outer area. This causes the appearance of mechanical differences in compression at the edges. This process occurs continuously so that the compression area will oscillate periodically to become a source of vibration. This vibration then propagates, vibrates the outside air, and travels as acoustic waves.

Figure 1 also shows the position of the vibration source line depending on the ratio of the length parameters *μ* and *d*. In the condition of *μ* < *d*, the position of the vibration source line will be in the first layer so that the photoacoustic signal intensity that appears will be dominantly influenced by the characteristics of the first layer material only. This signal has a relation in the form

(3) $$Q_{1}\propto \frac{\alpha_{1}^{1/2}}{2\pi \;f_{\mathrm{mod}}\;k_{1}^{2}}$$

*Q_1_, α_1_* and *k_1_* are the proportional photoacoustic signal intensity, the thermal diffusivity and thermal conductivity of the first layer respectively. If the thickness of the second layer increases (e.g., due to a foreign body), then *μ* > *d* can occur, resulting in a change in the intensity of the photoacoustic amplitude to

(4) $$Q_{2}\propto \frac{\alpha_{2}^{1/2}}{2\pi \;f_{\mathrm{mod}}\;k_{2}^{2}}$$

*α_2_* and *k_2_* are the thermal diffusivity and thermal conductivity of the second layer, respectively. Furthermore, using Equations 3 and 4, foreign objects under the surface can be detected, namely, through changes in the intensity of the photoacoustic signals that occur *Q_1_* and *Q_2_*.

### 2.2 Photoacoustic imaging systems

**FIGURE 2:**
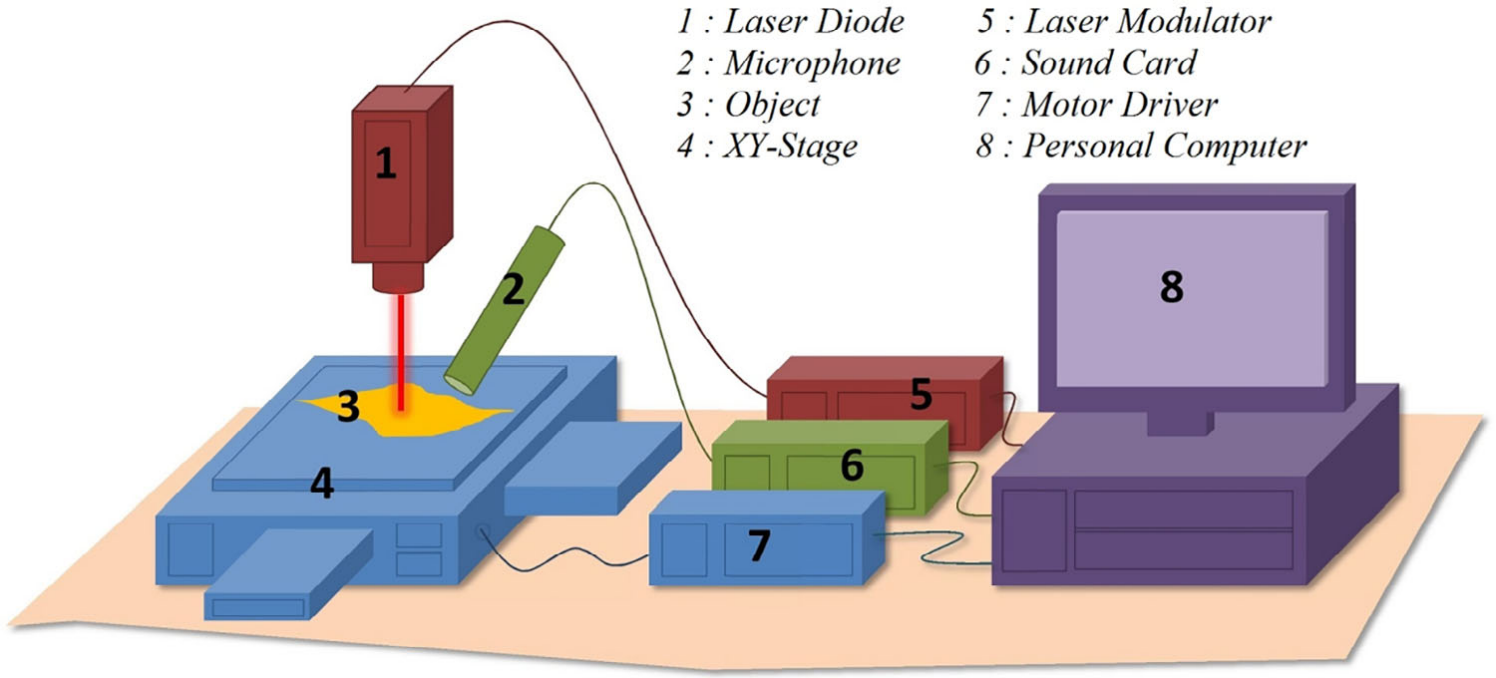
Scheme of the experimental setup, which consists of several main parts, namely, the xy‐stage, the laser diode, and the microphone. The microphone is placed at a distance of 3 mm from the platform so that it does not touch the object.

The system consists of a number of main parts, namely, the xy‐stage, laser diode, and microphone. The experimental setup is shown in Figure 2. The object is located on a computer‐controlled x‐y drive. The computer communicates with the Computerized Numerical Control (CNC) positioner (Trocen, AWC7813 Laser Motion Controller) to place the laser spot at coordinates (1,1). The laser is then turned on and the microphone records the acoustic signal that appears. The laser moves with a predetermined time and this process continues until it reaches the last position, which is coordinate (50,50). This audio recording data is then partitioned to form a photoacoustic image. Each coordinate is recorded for 1/100 min (about 0.6 s) so that the total time required is 25 min. Furthermore, acoustic recording has been carried out using Audacity software (Audacityteam.org, cross‐platform free audio software).

The laser diode is modulated at a certain frequency to achieve the expected thermal diffusion length. Furthermore, the laser beam hits the sample so that it vibrates and produces acoustic waves. These waves are then sensed by the microphone, and the data are sent via the soundcard to the computer. Using the Fourier transform, the data are processed, and the acoustic intensity is measured. This is conducted repeatedly for each x,y coordinate in the planned measurement area. After the entire area has been successfully scanned, the acoustic intensity data are then compiled into a photoacoustic image.

**FIGURE 3:**
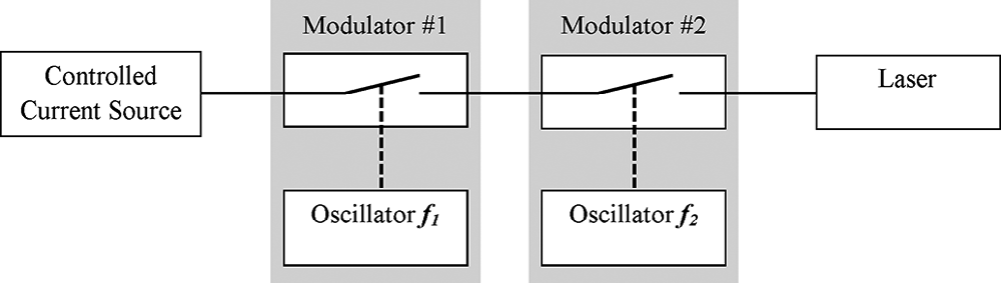
Dual modulator design to adjust the thermal diffusion length (first modulator with f_1_ frequency) and the laser power (second modulator with f_2_ frequency).

For the measurements, a piece of chicken meat (Cobb Broiler Strain) of 3 cm × 4 cm was used. The chicken meat sample was punctured with a 0.7 mm diameter syringe needle (OneMed Disposable Syringe, 22G × 1.5 inches) at a depth of approximately 0.3 mm. A scanning procedure was performed in the vicinity of the needle entry point, covering an area of 5 mm × 5 mm, as depicted in Figure 3a. To facilitate the imaging process, a diode laser (Roithner RLT9810G, 980 nm) was employed with modulation at a frequency of 100 Hz. The resulting acoustic signals were captured using a condenser microphone. The microphone used is Behringer's Ultra‐Linear Measurement Condenser Microphone (ECM8000) with a bandwidth of 20‐20 kHz and a sensitivity of 15 mV/Pa. Next, GNU Octave software (Octave.org, free software) was used to calculate the amplitude of the photoacoustic signal. This calculation was done for each segment using the Fast Fourier Transform (FFT) algorithm. FFT was used to calculate the amplitude intensity at the laser modulation frequency of 100 Hz. Furthermore, each audio intensity data is arranged following its pixel coordinates so that a photoacoustic image is formed.

### 2.3 Dual modulator controller

In this section, the first modulation adjusts the thermal diffusion length, while the second modulation reduces the laser power, as shown in Figure 3. Referring to the American National Standards Institute (ANSI) Z136.1,^33^ the maximum allowable laser energy can be calculated using the Maximum Permissible Exposure (MPE) equation

(5) $$\mathrm{MP}E_{skin}=2.0C_{A}\;\mathrm{for}\;0.400\leq \lambda <1.400\;\left(\mathrm{mW}/mm^{2}\right)$$

with

$$C_{A}=10^{2\left(\lambda -0.700\right)}\;\mathrm{for}\;0.700\leq \lambda <1.050$$

To meet safety standards according to ANSI, in this experiment, the maximum laser irradiance was set below 100 mJ/cm^2^ for a laser wavelength of 980 nm at 1‐s exposure, using a laser power meter (SANWA LP‐1).^34^ Safety glasses were used to protect the eyes from exposure to infrared laser light (ThorLab, LG7 Laser Safety Glasses). The experiment was conducted in a 3 m × 3 m closed room with a temperature controller (Omron, E5CC‐RX2ASM‐800 Temperature Controller) to prevent unplanned laser exposure while keeping the operational temperature stable.

## 3 RESULTS

**FIGURE 4:**
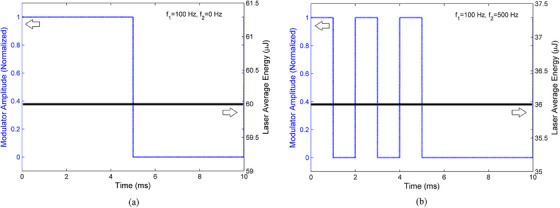
The effect of using second modulator to adjust the laser energy, before (a) and after (b). At a frequency of f_2_ = 500 Hz, there is a decrease in energy from 60 μJ close to the MPE limit of 36 μJ for each mm^−2^.

Using Equation 5, if the laser wavelength used is 0.980 μm, the CA constant value can be calculated as 3.63 so that the MPE_skin_ value is 7.26 mW.mm^−2^. Then, according to the calculation of the thermal diffusion length in the previous section, a frequency of 100 Hz or 10 ms modulation is obtained. With a 50% duty cycle, the laser exposure time for each cycle is 5 ms, so that the MPE energy density value of 36 μJ.mm^−2^ is obtained. Furthermore, using the RLT9810G laser datasheet, the laser energy density can be calculated to be 60 μJ.mm^−2^, as shown in Table 1. This value is almost double the allowable MPE limit. Therefore, it needs to be lowered. The strategy used as described earlier is to reduce the laser exposure time while maintaining the fundamental frequency corresponding to the thermal diffusion length. Figure 4 shows the process of changing the laser energy when given the second modulation of 500 Hz.

**TABLE 1:** Laser parameters and energy density calculation.

Parameters		
Power	12	mW
Energy (5 ms)	60	μJ
Beam divergence	0.52	rad
Distance	0.21	cm
Cross‐sectional area	0.99	mm^2^
Energy density	60	μJ.mm^−2^

**FIGURE 5:**
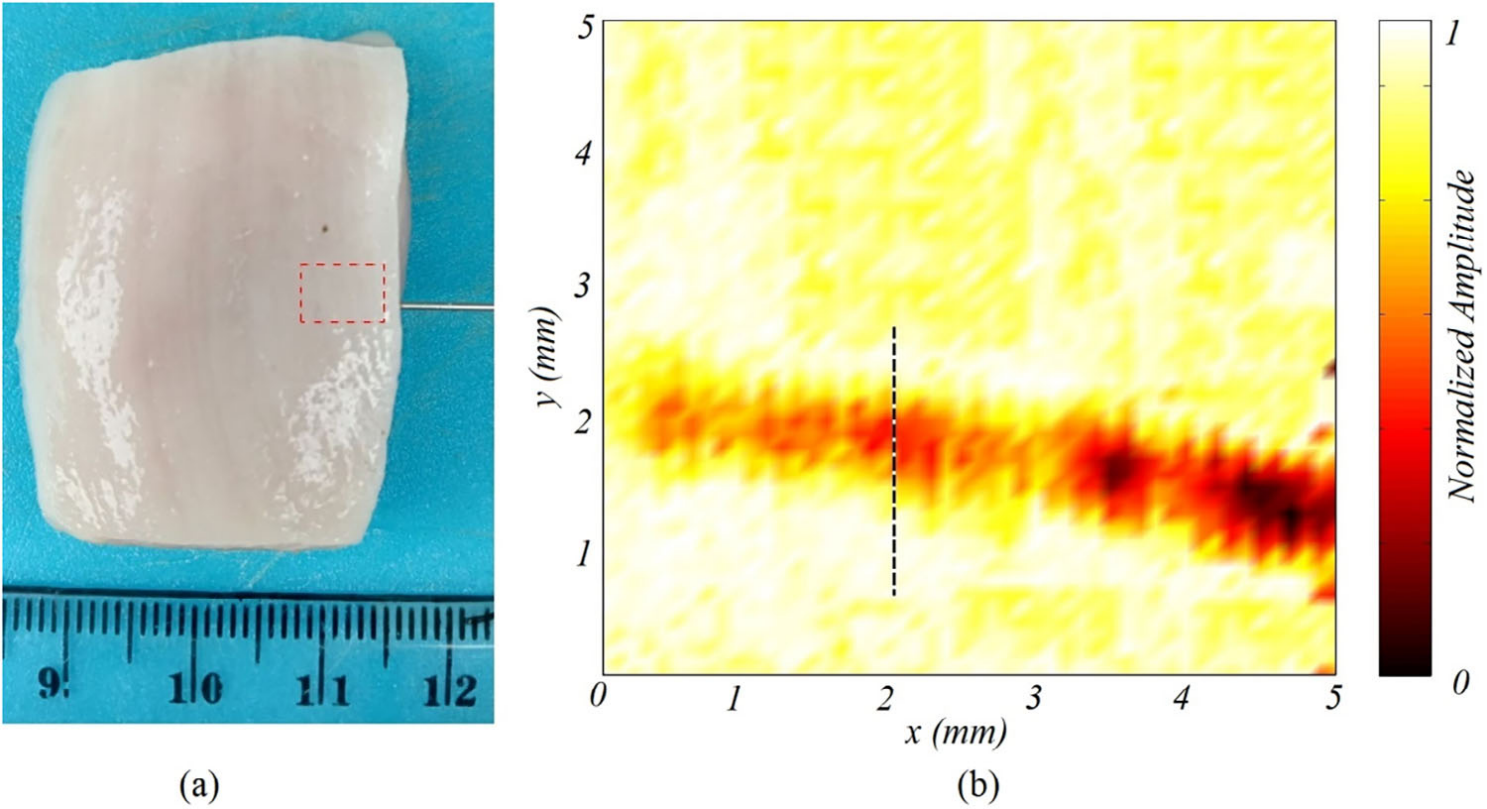
(a) A piece of chicken meat punctured by a needle as a foreign body model, the photoacoustic image generated from scanning the surface area of the chicken meat (marked with a red box), (b) the dotted line at x = 2 mm is the ROI (Research of Interest) for the next analysis.

The average peak measurement of the photoacoustic signal is at −20 dB or equivalent to a pressure of 2 μPa. According to the Audacity operation manual, if the 16‐bit Pulse Code Modulation‐Waveform Audio File Format (PCM‐WAV) recording type is selected, uncompressed WAV format data of about 5 MB per minute will be generated. If the movement of the laser from the initial coordinates to the end is taken for 25 min, a 125 MB file is generated for each photoacoustic image data. In the recording process using Audacity, the sampling rate was set at 44.1 kHz. Therefore, within 25 min, there would be 44 100 × 25 × 60 samples or 66 150 000 samples. To form a 50 × 50 pixel image, the total samples are divided into 2500 segments where each segment represents the intensity of 1 pixel. From this calculation, 26 460 samples are be calculated for each segment. The calculated results for each data point are assigned as pixel intensities, forming a photoacoustic image as illustrated in Figure 5b.

## 4 DISCUSSION

**FIGURE 6:**
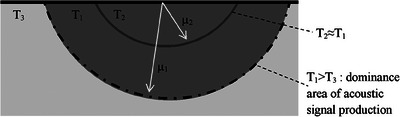
Thermal distribution in dual modulation laser, acoustic signal production will be dominated by the outer spherical skin area due to the greater temperature difference so that a stronger expansion difference appears.

In the laser energy setting section using a dual modulator, the second modulation will make the total laser exposure time decrease from 5 to 3 ms for each cycle, thus meeting the allowable MPE limit. In terms of thermal diffusion for frequencies of 100 and 500 Hz, it will have a range of 0.55  and 0.11 mm, respectively. If the length of thermal diffusion is considered as the radius of a thermal sphere, the second thermal diffusion length will be located in the first thermal distribution sphere. While the dominance of acoustic signal production will occur at the edge of the large thermal sphere where the temperature difference is more significant. A stronger mechanical expansion difference appears (Figure 6). Thus, even though mixed modulation of 100 and 500 Hz is carried out, the acoustic signal produced will still be dominated by the influence of the lower frequency of 100 Hz. This developed method works as expected. It reduces the laser energy density but still maintains the base frequency thermal diffusion range.

In the generated photoacoustic image, the position of the syringe is clearly discernible, with the needle displaying a relatively lower pixel intensity compared to its surrounding area Figure 5b. Using the object parameter data in Table 2 and Equation 6, the thermal diffusivity for meat and syringe needle is 96.6×10^−6^ m^2^/s and 3.98×10^−6^ m^2^/s, respectively. Furthermore, using Equation 2, the thermal diffusion length at 100 Hz laser modulation for chicken meat can be calculated as 0.55 mm.

(6) $$\alpha_{1}=\frac{k_{1}}{\rho C_{p}}$$

**TABLE 2:** Physical and thermal parameters of the objects used in this model.^35^, ^36^

Parameter	Chicken meat	Syringe needle
Thermal conductivity *k* (W/m.K)	0.4	15
Density *ρ* (kg/m^3^)	1.15	7500
Spesific heat *Cp* (J/kg.K)	3600	502

**FIGURE 7:**
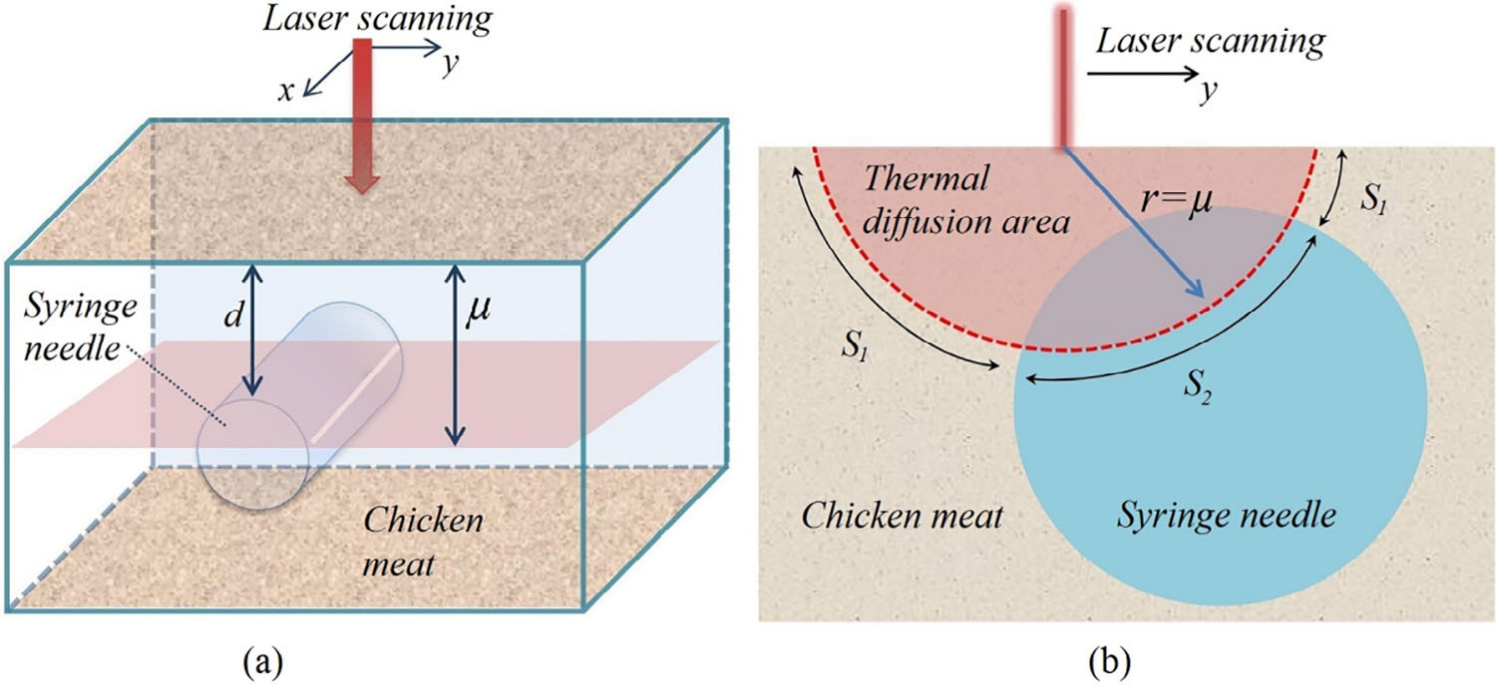
(a) Illustration of syringe needle in meat as foreign body model, (b) cross‐sectional image used for calculation of proportional photoacoustic signal intensity *Q_model_* .

In this experiment, the needle is punctured at a depth of 0.3 mm so that the thermal diffusion length can reach the syringe needle. To calculate the proportional photoacoustic signal intensity, an illustration of Figure 7b is used, where the scanning direction is taken in the y‐axis direction.

Using this illustration, the thermal diffusion area will centre on the laser point and shift towards the syringe needle. When the thermal diffusion area and syringe needle interact, regions *S_1_* and *S_2_* will appear. As explained in Equations 3 and 4, *S_1_* is the area that produces amplitude *Q_1_* while *S_2_* is the amplitude generator *Q_2_*. Furthermore, because the syringe needle is cylindrical while the thermal diffusion is spherical, when the laser point shifts, the area of interaction *S_1_* and *S_2_* changes non‐linearly. Therefore, to estimate the photoacoustic amplitude of the *Q_model_*, the following equation is used

(7) $$Q_{\text{model}}=Q_{1}\underset{r=\mu }{\;\int \int \;}dS_{1}+Q_{2}\underset{r=\mu }{\;\int \int \;}dS_{2}$$

which is the sum of the amplitudes of *Q_1_* and *Q_2_* multiplied by the area of the interaction area *S_1_* and *S_2_*. In this case, the sphere integral is used to calculate the area that is part of *S_1_* and *S_2_*.

**FIGURE 8:**
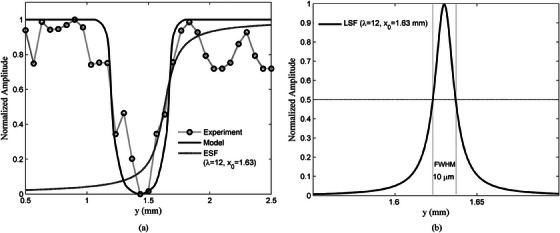
(a) Comparison curve of proportional photoacoustic signal intensity of experimental results and the model, experimental data taken from the ROI of Figure 5b (dashed vertical line at x = 2 mm). (b) The results of fitting the data with the ESF and calculating the FWHM with the LSF curve resulted in a spatial resolution of 10 μm.

Figure 8a presents a comparison between the experimental and modelled data using Equation 7 and the Region of Interest (ROI) depicted in Figure 8b. The modelled equation demonstrates the ability to accurately track the variations in the amplitude curve. The slight fluctuation observed at the peak of the experimental curve is attributed to the surface roughness of the chicken meat. Additionally, the s (MAPE) calculation between the model and the experiment yielded a value of 11%, indicating a relatively small deviation between the two.

To calculate the spatial resolution of the imaging system image, the equation is used

(8) $$ESF\left(x\right)=\frac{1}{2}+\frac{1}{\pi }\tan^{-1}\left(\lambda \left(x-x_{0}\right)\right)$$

where *x*, *x_0_*, and *λ* are the pixel position, curve centre position, and fitting parameter variables, respectively.^37^, ^38^ The ESF is then derived into the LSF equation and the spatial resolution of an image can be calculated from the Full‐Width Half Maximum (FWHM) of the LSF curve by the equation

(9) LSFx=λλππ1+λ2x−x02

Figure 8 shows the curve matching process ESF (a) and LSF (b). The calculation results show an image spatial resolution of 10 μm.

## 5 CONCLUSION

A successful development of a non‐contact photoacoustic imaging system has enabled the visualization of a syringe needle within meat as a foreign object with 10 μm spatial resolution. This system utilizes the principles of thermally thin and thick solid conditions based on the Rosencwaig‐Gersho photoacoustic theory, allowing for clear imaging of subsurface objects from the surface of the material. Experimental results also demonstrate the efficacy of the non‐contact photoacoustic signal intensity measurement method, employing audio frequencies to detect foreign objects embedded in chicken meat. Additionally, a mathematical model was successfully established to comprehend the process of photoacoustic signal formation, with a MAPE difference of 11% compared to the experimental findings. Dual modulator successfully regulates laser energy at MPE limit. With a method based on measuring amplitude intensity at audio frequencies, the detector does not require high speed. In terms of instrumentation, this makes PA imaging systems easier to build and apply using existing commercial audio devices. This technique provides opportunities for the application of photoacoustic methods on portable devices such as smart‐phones that already have audio recording facilities.

## AUTHOR CONTRIBUTIONS

**Andreas Setiawan**: Data collection; modeling; result validation; writing—original draft preparation and editing. **Chia‐Yi Huang**: Reviewing. **Mitrayana Mitrayana**: Conceptualization; supervision; result validation; reviewing. All authors discussed the result and commented on the manuscript.

## CONFLICT OF INTEREST STATEMENT

The authors declare no conflicts of interest.
